# The Emerging Role of Sfrp5 and Wnt5a in the Pathogenesis of Obesity: Implications for a Healthy Diet and Lifestyle

**DOI:** 10.3390/nu13072459

**Published:** 2021-07-19

**Authors:** Diamanto Koutaki, Athanasios Michos, Flora Bacopoulou, Evangelia Charmandari

**Affiliations:** 1 Division of Endocrinology, Metabolism and Diabetes, First Department of Pediatrics, National and Kapodistrian University of Athens Medical School, “Aghia Sophia” Children’s Hospital, 11527 Athens, Greece; dkoutaki@med.uoa.gr; 2 Division of Infectious Diseases, First Department of Pediatrics, National and Kapodistrian University of Athens Medical School, “Aghia Sophia” Children’s Hospital, 11527 Athens, Greece; achos@med.uoa.gr; 3 University Research Institute of Maternal and Child Health & Precision Medicine, and UNESCO Chair on Adolescent Health Care, National and Kapodistrian University of Athens, “Aghia Sophia” Children’s Hospital, 11527 Athens, Greece; bacopouf@hotmail.com; 4 Division of Endocrinology and Metabolism, Center of Clinical, Experimental Surgery and Translational Research, Biomedical Research Foundation of the Academy of Athens, 11527 Athens, Greece

**Keywords:** SFRP5, Wnt5a, overweight, obesity, childhood, metabolic

## Abstract

In recent decades, the prevalence of obesity has risen dramatically worldwide among all age groups. Obesity is characterized by excess fat accumulation and chronic low-grade inflammation. The adipose tissue functions as a metabolically active endocrine organ secreting adipokines. A novel duo of adipokines, the anti-inflammatory secreted frizzled-related protein 5 (Sfrp5) and the proinflammatory wingless type mouse mammary tumor virus (MMTV) integration site family member 5A (Wnt5a), signal via the non-canonical Wnt pathway. Recent evidence suggests that Sfpr5 and Wnt5a play a key role in the pathogenesis of obesity and its metabolic complications. This review summarizes the current knowledge on the novel regulatory system of anti-inflammatory Sfrp5 and pro-inflammatory Wnt5a, and their relation to obesity and obesity-related complications. Future studies are required to investigate the potential role of Sfrp5 and Wnt5a as biomarkers for monitoring the response to lifestyle interventions and for predicting the development of cardiometabolic risk factors. These adipokines may also serve as novel therapeutic targets for obesity-related disorders.

## 1. Introduction

Obesity has emerged as a pandemic of the 21st century, and its global prevalence has nearly tripled since 1975 [1]. In 2016, approximately 39% of adults older than 18 years were overweight and 13% were obese. According to a simulation model based on the current obesity trends, it is estimated that by 2030 an additional 65 million adults in the USA and 11 million adults in the UK will have obesity [2]. Overweight and obesity account for the increased morbidity and mortality, as well as for an increased financial burden on health systems.

Obesity in childhood and adolescence also represents a major health problem of our century. Data of the body mass index (BMI) from 200 countries indicate that the prevalence of obesity in children and adolescents has increased rapidly, from 4% in 1975 to 18% in 2016 [3]. In addition, according to the World Health Organization (WHO), in 2016, more than 41 million children under the age of 5 years and 340 million children and adolescents aged 5–19 years were overweight or obese [4]. In the United States, an obese child is estimated to cost the health care system during his/her lifetime an average of $19,000 more than a child of normal BMI [5]. Therefore, one of the main global targets of the WHO is to curb the growth of overweight and obesity by 2025 [6]. To this end, the early implementation of multidisciplinary personalized intervention programs for the prevention and management of overweight and obesity in childhood and adolescence is imperative. Several lifestyle intervention strategies have been proposed, which mostly involve a healthy lifestyle of diet, sleep and physical activity [7,8,9]. In addition, novel e-health applications have been developed to address the epidemic of childhood obesity [10,11].

Obesity is a multifactorial, complex disease with genetic, environmental and socio-economic factors implicated in its etiology [12,13,14,15]. It is characterized by the increased accumulation of body fat, especially visceral adipose tissue. BMI (body weight in kilograms divided by the square of height in meters) is used as the standard clinical measure of overweight and obesity, although it has limitations when it is applied for muscular subjects, given that it cannot distinguish between lean and fat mass and fat mass distribution [8,9]. According to WHO, for adults, overweight corresponds to a BMI between 25–30 kg/m^2^ and obesity to a BMI > 30 kg/m^2^. In children and adolescents, these limits vary considerably according to age and gender. Thus, children and adolescents with a BMI ≥ 85^th^ percentile for age and gender are classified as overweight, while those with a BMI ≥ 95^th^ percentile as obese.

Obesity is associated with a state of chronic, low-grade inflammation in adipose tissue, and an increased risk for the development of diabetes mellitus type 2 (T2D), dyslipidemia, cardiovascular disease and certain types of malignancies [15,16,17]. Childhood obesity also predisposes individuals to several diseases, which affect almost every system in the human body. These diseases, although traditionally considered as diseases of ‘adults’, are nowadays rapidly emerging in the pediatric population. In particular, obese children are at higher risk for hyperinsulinemia, insulin resistance, prediabetes and T2D [18]. Moreover, there is an increased incidence of metabolic syndrome, atherosclerotic cardiovascular disease and hypertension [19]. Non-alcoholic fatty liver disease (NAFLD) is strongly associated with obesity, and represents the most common cause of liver disease during childhood [20]. Finally, obese subjects are more prone to develop hyperandrogenemia, polycystic ovary syndrome, obstructive sleep apnea, musculoskeletal problems, skin lesions, such as acanthosis nigricans, idiopathic intracranial hypertension and psychological implications, such as anxiety, depression and low self-esteem [21,22,23,24,25,26].

The adipose tissue has long been recognized as an active endocrine organ secreting multiple bioactive substances, including chemokines, cytokines and hormones [27]. Taking into account its size, it represents the largest endocrine gland in the human body. These bioactive substances are known as adipocytokines or adipokines. Adipokines are mediators, which act via autocrine, paracrine and endocrine signaling pathways, and exert pro-inflammatory or anti-inflammatory actions.

The adipose tissue matrix is comprised of adipocytes, as well as other cell types, such as progenitor cells, immune cells and endothelial cells that secrete even more mediators [28]. In response to the positive energy balance caused by obesity, severe adipose tissue dysfunction is observed. Adipocytes increase in number (hyperplasia) and enlarge in size (hypertrophy). Excess free fatty acids lead to ectopic lipid accumulation and contribute towards the development of obesity-related metabolic disturbances, especially insulin resistance [29]. This dysregulation is accompanied by an increase in the secretion of various proinflammatory chemokines and cytokines by macrophages, causing chronic low-grade inflammation [29,30], which in turn triggers a multitude of complications [31]. The imbalance of pro-inflammatory and anti-inflammatory adipokines plays a prominent role in the pathogenesis of obesity-related complications by acting in almost every system of the human body. Ample research studies have been conducted on adipocyte-derived factors, such as the anti-inflammatory adiponectin and IL-10, and the pro-inflammatory leptin, resistin, visfatin and ghrelin. Since then, an expanding number of more than 600 adipokines have been identified [32]. In 2010, Ouchi et al. first described the role of the novel anti-inflammatory adipokine Sfrp5 (secreted frizzled related protein) in inhibiting the proinflammatory adipokine Wnt5a (wingless type mouse mammary tumor virus (MMTV) integration site family member 5A) [33]. Subsequently, many studies have tried to elucidate the mechanisms through which Sfrp5 and Wnt5a are associated with obesity and cardiometabolic risk factors, as well as their potential role in developing new pharmaceutical compounds. The purpose of this review is to summarize the current knowledge on the emerging role of Sfrp5 and Wnt5a in the pathogenesis of obesity and its comorbidities both in adults and children.

## 2. Adipokines Sfrp5 (Secreted Frizzled Related Protein) and Wnt5a (Wingless-Related Integration Site 5A)

A newly recognized duo of adipokines is Sfrp5 and Wnt5a [33]. WNT (wingless-related integration site) proteins are a family of 19 extracellular glycoproteins in the human species with paracrine and autocrine action [34]. These proteins regulate biological processes, such as cell differentiation, proliferation, polarity and migration, as well as body functions, such as embryogenesis and development of the cardiovascular system. They act on their target cells through binding to the extracellular cysteine-rich domain of frizzled (Frz) receptors and are inhibited by Dickkopf (Dkk) and SFRP proteins in the extracellular space [35]. WNT signaling consists of two main pathways: canonical/β-catenin-dependent and non-canonical/β-catenin-independent. Thus, most Wnt proteins (Wnt1, Wnt3a, Wnt10b, etc.) activate the canonical pathway, while some (mainly Wnt5a and Wnt11) activate the non-canonical pathway [36]. Canonical signaling stabilizes the cytoplasmic protein β-catenin, leading to increased transcription of target genes, and inhibits the transcription factor PPARγ (peroxisome proliferator-activated receptor gamma), which is responsible for the differentiation of progenitor cells into mature adipocytes [37]. Consequently, the storage function of mature adipocytes is limited and lipids are diverted to the liver and muscles, leading to various metabolic complications of obesity. The non-canonical signaling pathway is mainly related to insulin resistance and endothelial inflammation [34]. It is divided into two additional pathways, the non-canonical/Ca^2+^ pathway and the non-canonical/planar cell polarity pathway (PCP). The adipokines Sfrp5 and Wnt5a are involved in the non-canonical WNT/Ca^2+^ pathway.

Wnt5a acts as a proinflammatory adipokine and is expressed in adipose tissue, as well as in macrophages and CD14+ monocytes. It serves a key role in the formation of the innate immune response and inflammation [38]. Experimental results from rodent models indicate that its expression increases proportionally to obesity [33]. It exerts its actions by activating c-Jun N-terminal kinase 1 (JNK1) in macrophages and adipocytes. In adipocytes, activated JNK1 blocks the activity of IRS-1 (insulin receptor substrate-1), leading to reduced insulin signaling and the development of insulin resistance [39,40]. Thus, increased concentrations of Wnt5a lead to inflammation and impaired glucose tolerance. Finally, it has been associated with oxidative stress and atherosclerotic cardiovascular disease [41], as evidenced by the increased concentrations of Wnt5a in atherosclerotic plaques of mice and humans [42]. Pre-atherosclerotic lesions are caused by a potentiation of NADPH oxidase (nicotinamide adenine dinucleotide phosphate oxidase) by Wnt5a and, consequently, the production of reactive oxygen species (ROS). Excess ROS facilitate the migration of vascular wall smooth muscle cells, a process directly associated with the formation of atherosclerotic plaques via a pathway involving the enzyme USP17 (deubiquitinating enzyme ubiquitin-specific protease 17) and GTPase RAC1 [41].

Sfrp5 has been recently identified as an adipokine with insulin sensitizing and anti-inflammatory properties, which is secreted by healthy adipocytes [33]. In humans, five members of Sfrp proteins are produced in several tissues, such as the visceral, subcutaneous and pericardial adipose tissue, the liver, the heart, mononuclear blood cells and the pancreas [43]. SFRPs are soluble proteins of approximately 300 amino acids consisting of a N-terminal cysteine-rich domain (CRD), which is highly homologous to wingless-type (Wnt) receptor frizzled proteins, and a small hydrophilic C-terminal domain [34]. Sfrp5 binds to Wnt5a extra-cellularly and inhibits its interaction with the Frz receptor, thus mitigating the inflammation caused by Wnt5a. A study in obese mice suggests that Sfrp5 is highly expressed in normal adipose tissue but decreases with weight gain [33]. Targeted mutation of Sfrp5 in animal models induces insulin resistance, glucose intolerance, and hepatosteatosis when the animals are fed a high-fat diet [44]. In addition, it reduces liver fibrosis through Wnt5a/Fz2 signaling, while also acting protectively on the pancreas [45]. Rats not expressing Sfrp5 show proliferation of pancreatic β-cells via IGFBP-3, an insulin-compensatory lesion observed in obesity [46,47]. Furthermore, it is involved in the process of maturation of fat cells, as the Sfrp5 gene is a target of the transcription factor PPARγ [48]. Finally, it exerts vasodilating actions by regulating nitric oxide production via inhibiting the WNT5A/JNK pathway in endothelial cells [49]. Therefore, Sfrp5 plays a pivotal role in the metabolic complications of obesity by inhibiting Wnt5a and the inflammation that the latter promotes. 

Wnt signaling plays a crucial role in regulating adipogenesis. Wnt proteins hinder adipogenesis by suppressing PPARγ and thereby lipid accumulation [50]. Studies indicate that Wnt5a may inhibit adipogenesis [51,52], while Sfrp5 promotes it and acts as a marker of mature adipocytes [53,54]. Likewise, Sfrp5 may be a target-gene under the transcriptional regulation of pre-adipogenic transcription factor PPARγ [48]. Adipose tissue from Sfrp5-deficient mice exhibited decreased number of large fat cells, but a similar number of adipocytes compared to wild-type mice, indicating that Sfrp5 affects adipocyte hypertrophy rather than proliferation [55]. Furthermore, Sfrp5 possesses anti-inflammatory properties and may regulate adipose tissue inflammation. Macrophage infiltration is considered a marker of adipose tissue inflammation as macrophages encircle necrotic adipocytes, forming characteristic crown-like structures (CLCs) [56]. Fat biopsy specimens of obese individuals with macrophage CLSs displayed lower Sfrp5 expression compared with obese individuals, who were negative for CLS [33].

Wnt5a and Sfrp5 appear to be critical players in the regulation of systemic inflammation in obesity and glucose homeostasis, as well as mediators between adipose tissue and other key metabolic organs, including the liver and the pancreas.

## 3. Adult Subjects

### 3.1. Obesity

Obesity is associated with chronic low-grade inflammation and the dysregulation of adipokines plays an important role in promoting it. Therefore, it is hypothesized that Sfrp5 and Wnt5a may also be involved in the pathogenesis of obesity and obesity-related disorders. Clinical studies have been conducted to delineate the role of Sfrp5 and Wnt5a in obesity, and the regulation of their secretion in an obesogenic environment. In both obese, leptin-deficient (ob/ob) mice and wild-type (WT) mice, which were fed a high fat/high sucrose diet for 24 weeks, Sfrp5 expression was reduced and Wnt5a increased, while the ratio of Wnt5a/Sfrp5 was higher [33]. Moreover, in visceral fat biopsy specimens of obese individuals, tissue inflammation, indicated by CLS, was associated with lower Sfrp5 expression, compared to obese individuals without CLSs. Taken together, these results suggest that Sfrp5 regulation is related to obesity. Similarly, cross-sectional studies showed that Sfrp5 is negatively correlated with indices of obesity, such as BMI, waist-to-hip ratio (WHR), percent body fat, glycolipid metabolism and inflammation [41,57,58,59,60]. Interestingly, two large studies of primarily overweight and obese subjects suggested that Sfrp5 was negatively associated with adiposity, given that the progressive increase in body fat percentage over time was associated with a decrease in Sfrp5 concentrations [61,62]. Moreover, weight loss altered the expression of Wnt5 and Sfrp5, leading to increased Sfrp5 and decreased Wnt5a expression levels [53,63]. A large observational study that evaluated the diet in 1128 volunteers, demonstrated that high consumption of sugar-sweetened beverages along with low fruit and vegetable intake resulted in lower Sfrp5 concentrations, suggesting a dietary modification of Sfrp5 [64]. This result is in accordance with the reported effect of a HF/HS diet in lower Sfrp5 expression in obese rodents [33]. Further studies provided additional evidence that Wnt5a is important in linking inflammation to metabolism, and that nutrition might prevent and/or treat Wnt5a-mediated metabolic inflammation [65]. Although sufficient evidence suggests upregulation of Wnt5a and downregulation of Sfrp5 in obesity, several studies have shown conflicting results. Further research is required to delineate how these factors are regulated by excess fat and how they are modified in relation to body mass change.

### 3.2. Insulin Resistance 

The rising prevalence of obesity globally is accompanied by an increase in the prevalence of T2D [66]. The link between obesity and diabetes is reflected in common pathways of pathogenesis, with inflammation and adipokines serving an essential role in both disorders. In this aspect, several studies have focused on the impact of the Wnt5a/Sfrp5 complex on glucose metabolism. Data from Asian and European populations demonstrated that higher circulating Sfrp5 concentrations were associated with lower odds of prediabetes/T2D [57,58,60,61]. Furthermore, Sfrp5 was inversely correlated with HOMA-IR, HbA1C and insulin concentrations, while oral glucose intake led to a rapid and significant suppression of circulating Sfrp5 concentrations. In patients with metabolic syndrome (MetS), circulating Sfrp5 concentrations were lower than in control subjects, they progressively decreased as the MetS components increased, and were negatively correlated with HbA1C and insulin resistance (HOMA-IR) [59]. A cutoff value for circulating Sfrp5 of 46.78 μg/L was proposed to predict MetS development (sensitivity 70.1%, specificity 47.8%). In patients with T2D and latent autoimmune diabetes (LADA), circulating Sfrp5 concentrations were significantly lower than in healthy control subjects, although no differences were observed between LADA and T2D groups. Moreover, Sfrp5 was negatively correlated with HOMA-IR, diabetes duration and BMI [67]. These results support a role for Sfrp5 as a protective factor in the pathogenesis of autoimmune diabetes.

On the other hand, subjects with T2D had higher Wnt5a concentrations, and Wnt5a concentrations were positively correlated with fasting plasma glucose, IL-6 and triglyceride concentrations [65]. Wnt5a concentrations were not influenced by common single-nucleotide polymorphisms in the human Wnt5a gene; however, environmental factors influenced the concentrations of Wnt5a significantly. More specifically, Wnt5a concentrations were low in subjects with high nutritional load of long-chain eicosatetraenoic acid, as well as in those with increased gut microbiome α diversity. In vitro studies showed that stimulation of the IL-6 receptor or the long-chain fatty acid receptor GPR40 influenced the expression of Wnt5a in human macrophages [65].

Insulin resistance is recognized as a prevalent pathogenetic factor in the development of polycystic ovarian syndrome (PCOS). Research studies on Sfrp5 and Wnt5a in women with PCOS have yielded contradictory results. The majority of studies suggest a negative association between Sfrp5 and insulin resistance, as indicated by the lower concentrations of Sfrp5 in PCOS women [57].

Another hypothesis that has been investigated is whether antidiabetic drugs, such as metformin, liraglutide and rosiglitazone, improve insulin sensitivity by regulating the Sfrp5/Wnt5a axis. Metformin inhibits liver gluconeogenesis and decreases hepatic glucose output. Liraglutide is a glucagon-like peptide-1 (GLP-1) analogue, while rosiglitazone belongs to thiazolidinediones (TZDs), a class of antidiabetic drugs that increase glucose utilization and improve peripheral tissue sensitivity. In 3T3-L1 pre-adipocytes treated with rosiglitazone and metformin, Sfrp5 mRNA expression in mature adipocytes increased by 34% and 19%, respectively, while Sfrp5 protein secretion increased by 10% and 6%, respectively [68]. Patients with T2D treated with liraglutide once daily for 12 weeks, demonstrated increased Sfrp5 concentrations compared with the placebo group [57]. Similarly, 12-week treatment with metformin resulted in a significant increase in Sfrp5 concentrations and improvement of insulin sensitivity [60]. Metformin enhances GLP1 secretion via a cross talk between insulin and Wnt signaling [69].

The variations noted in the results of the above studies may be attributed to differences in both the severity and duration of diabetes, and hence differences in inflammatory status, differences in the genetic background, as evidenced by differences in ethnicity, concomitant administration of other medications, the cross-sectional design of studies, as well as the occasional relatively small sample size.

### 3.3. Cardiovascular Disease

Cardiovascular disease remains one of the leading causes of mortality worldwide and its association with obesity is well established [15]. Inflammation constitutes a fundamental element of atherogenesis, and for this reason, the role of adipokines in the formation of atherosclerotic plaques has been thoroughly investigated. Akoumianakis et al. showed that Wnt5a induced oxidative stress in the arterial wall and contributed to vascular atherogenesis through a previously unknown pathway involving the enzyme USP17 [41]. In this prospect, reports have suggested the involvement of Wnt5a in endothelial dysfunction, especially in relation to insulin resistance [70,71]. In subjects with T2D, serum Sfrp5 concentrations were significantly and positively correlated with arterial stiffness, and were independently associated with brachial ankle pulse wave velocity, even after adjustment for potential confounders. In vitro studies showed that Sfrp5 exerted vasorelaxant actions and regulated NO through inhibition of WNT5A/JNK signaling in vascular endothelial cells. Treatment with Sfrp5 restored the Wnt5a-induced impaired vasorelaxation in a dose-dependent manner in rat thoracic aorta via an endothelial NO synthase-dependent mechanism. In addition, treatment with Sfrp5 restored the Wnt5a-induced reduction of NO production via endothelial NO synthase in human endothelial cells, and ameliorated the Wnt5a-induced changes in the phosphorylation of JNK, AKT, and endothelial NO synthase. These data indicate that Sfrp5 may have a protective compensatory role against atherosclerosis under conditions of metabolic dysfunction [49]. In vitro data suggest that Sfrp5 may play a protective role in the process of atherosclerosis. In human aortic smooth muscle cells (VSMCs), Sfrp5 significantly suppressed HP-induced calcification and protected against oxidative stress-induced apoptosis in human aortic endothelial cells through inhibiting the Wnt/β catenin signalling pathway [72,73]. By suppressing the Wnt/β-catenin and p38/mitogen-activated protein kinase signaling pathways in Sprague Dawley rat aortas, Sfrp5 may inhibit smooth muscle cell proliferation, migration and inflammation [74]. Moreover, Sfrp5 might serve as a novel vasodilative adipokine through NO production by antagonizing the WNT5A/JNK signaling pathway in endothelial cells [49]. In rodent models, the genetic deficiency of Sfrp5 was associated with a greater myocardial infarct size following ischemic/reperfusion injury, greater apoptotic cell death of cardiac myocytes and greater degree of inflammation in the infarct zone [75]. Serum Sfrp5 concentrations in subjects with coronary artery disease (CAD) were significantly lower than those in non-CAD subjects and were inversely related to the severity of CAD and serum high sensitivity C-reactive protein (hs-CRP) concentrations [76]. Therefore, Sfrp5 could be a useful biomarker for the evaluation of subjects suspected of having CAD.

In the setting of acute ischemia, Sfrp5 concentrations were significantly higher in patients with acute ST-elevation myocardial infarction (STEMI) than in those without CAD, although they decreased over time [77]. In addition, high serum Sfrp5 concentrations at baseline were associated with a decreased risk of reduced left ventricular ejection fraction (LVEF) at 3 months, independent of peak hypersensitive cardiac troponin I (hs-cTnI) and baseline cardiac function [77]. These findings indicate a cardioprotective role of Sfrp5 in the setting of myocardial ischemic/reperfusion injury, given that higher baseline serum Sfrp5 concentrations were associated with a more favorable prognosis. They also suggest that Sfrp5 may be a potential therapeutic target in acute STEMI.

### 3.4. Non-Alcoholic Fatty Liver Disease/Liver Fibrosis

The prevalence of non-alcoholic fatty liver disease (NAFLD) has increased during the last decade in Western countries, and represents the most important cause of chronic liver disease [78]. Obesity is an independent risk factor for chronic liver disease and adipokine dysregulation contributes essentially in its pathogenesis [79]. Various adipokines, including leptin, adiponectin, TNF-alpha and resistin have been associated with liver disease. Considering this adipokine-liver cross-talk, a possible association between Sfrp5/Wnt5a and liver disease is hypothesized. Experimental evidence indicates that Sfrp5 decreases accumulation of triglyceride and induction of hepatic steatosis through a pathway involving secreted lymphocyte antigen-6/urokinase-type plasminogen activator receptor-related peptide (Slurp-1) [44]. Likewise, Sfrp5-deficient mice exhibited a greater degree of hepatic steatosis, with a higher triglyceride content when fed a high fat diet [33]. In vitro administration of Sfrp5 ameliorates hepatic lipid accumulation, inflammation and non-alcoholic steatohepatitis (NASH) [33,80]. As a pro-inflammatory cytokine, higher Wnt5a expression was found in animal models of liver fibrosis and its profibrotic properties were primarily regulated by the profibrogenic mediator TGF-β and activation of hepatic stellate cells [81,82,83,84]. Inhibition of Wnt5a/Fz2 signaling by Sfrp5 and subsequent hepatic stellate cells inactivation, mitigated mouse liver fibrosis [45]. In accordance to in vitro data, cross-sectional designed studies in obese human subjects support an increase in Wnt5a concentrations in NAFLD patients, although no significant association with serum sfrp5 was observed [63,85,86]. However, hepatic Sfrp5 protein levels were an independent predictor of NAFLD in a group of morbidly obese women, while, Sfrp5 mRNA and protein levels were lower in hepatocytes of non-alcoholic steatohepatitis (NASH) subjects compared to the control group (12). This result is in accordance with a recent study in L02 hepatocytes in which inhibition of the PPARγ/SFRP5 pathway promoted the conversion of NAFL into NASH by activation of macrophage Kupffer cells [87]. Serum Sfrp5 did not correlate with markers for the severity of NAFLD like hepatic steatosis index and liver enzymes [88].

These data provide evidence of a possible role of Wnt5a in NAFLD and liver fibrosis and their alleviation by Sfrp5. Additional human studies will be required to shed light on the influence of Wnt5a/Sfrp5 in NAFLD and hepatic fibrosis observed in obesity.

## 4. Children and Adolescents

Data on Sfrp5 and Wnt5a in the pediatric population have so far been limited. Prats-Puig et al. determined the concentrations of both these adipokines in 342 prepubertal children and investigated their association with metabolic markers. In addition, Sfrp5 and Wnt5a were studied further in conditioned media of adipose tissue explants obtained from 12 children. The concentrations of Sfrp5 and Wnt5a correlated positively in serum and in conditioned media. Lower circulating Sfrp5 concentrations were associated with higher BMI and lower concentrations of adiponectin. Circulating Wnt5a correlated strongly with insulin resistance and hepatic enzymes (SGOT, SGPT), particularly in children with lower circulating Sfrp5 concentrations. These data suggest that Sfrp5 and Wnt5a comprise a balanced duo that may regulate metabolic homeostasis in prepubertal children [89]. This observation could explain the failure to upregulate Sfrp5 and sequester Wnt5a in obesity that may lead to unrestrained proinflammatory actions of Wnt5a. Lower Sfrp5 serum concentrations were associated with a higher likelihood for obesity and obesity comorbidities, such as elevated blood pressure, carotid intima media thickness, dyslipidemia and inflammation.

In line with this study, a relation between the two molecules and excess weight, insulin resistance and inflammation, was supported by another cross-sectional study [90]. Sfrp5 concentrations were lower in obese subjects, especially in those with metabolic syndrome. However, no difference was found in Wnt5a between lean and obese children. This finding was attributed to a difficult detection of Wnt5a in human circulation due to its autocrine or paracrine action. In a subgroup of the study population, a family-based lifestyle intervention program was performed for 6 months. Significant weight loss led to increased Sfrp5 concentrations and improvement of metabolic markers. Accordingly, implementation of a lifestyle intervention program in obese children with hypertension resulted in a rapid decrease in Sfrp5 but no change in Wnt5a concentrations [91]. At baseline, obese children with hypertension had lower Sfrp5 and higher Wnt5a concentrations, whereas a reduction of BMI and blood pressure after 6 months resulted in increased Sfrp5 concentrations. Therefore, it is likely that Sfrp5 is more sensitive to lifestyle interventions, and by binding to Wnt5a, it alleviates its inflammatory actions.

Finally, one study investigated whether umbilical cord Sfrp5 concentrations correlate with selected maternal parameters and neonatal anthropometric measurements [71]. Sfrp5 concentrations were determined in the umbilical cord and maternal serum of mothers with excessive gestational weight gain (EGWG) and healthy control subjects. In the EGWG group, umbilical cord Sfrp5 concentrations were negatively correlated with BMI gain and positively correlated with maternal serum Sfrp5, HbA1C and lean tissue index. EGWG subjects also had lower Sfrp5 concentrations than the control group. These findings suggest an early exposure of the developing fetus to an imbalance of these adipokines and the subsequent development of metabolic complications [92].

## 5. Conclusions

Sfrp5 is an adipokine which may play a protective role against obesity-related insulin resistance and T2D by binding to Wnt5a and improving insulin sensitivity. This review summarizes the current evidence on the emerging role of Sfrp5 and Wnt5a in the pathogenesis of obesity, T2D and cardiovascular disease. Chronic low-grade inflammation is a common denominator for all obesity-related comorbidities. Thus, the cross-talk between adipokines and target organs contributes to the inflammation and the pathogenesis of obesity-related complications. Sfrp5, as a decoy receptor of Wnt5a, expresses its anti-inflammatory properties via the JNK1 pathway in macrophages and might lead to a more favorable metabolic phenotype. The Sfrp5/Wnt5a regulatory system is still relatively unexplored, however, current evidence indicates that it plays a pivotal role in obesity, even in childhood and adolescence. Further studies are required to elucidate the mechanisms underlying their metabolic actions. Prospective studies will improve our understanding of their role in weight loss. Future research in children is imperative, since children are not usually on medical therapy for the management of obesity and/or its complications, and provide us with an opportunity to study the role of these adipokines in the early stages of the pathogenesis of obesity. Further studies are also required to investigate the potential role of Sfrp5 and Wnt5a as biomarkers for monitoring the response to lifestyle interventions and for predicting the development of cardiometabolic risk factors. Finally, these adipokines may also serve as novel therapeutic targets for obesity-related disorders.

## Data Availability

Data sharing not applicable.
